# Maskless Surface Modification of Polyurethane Films by an Atmospheric Pressure He/O_2_ Plasma Microjet for Gelatin Immobilization

**DOI:** 10.3390/mi9040195

**Published:** 2018-04-20

**Authors:** Man Zhang, Yichuan Dai, Li Wen, Hai Wang, Jiaru Chu

**Affiliations:** 1 Department of Precision Machinery and Instrumentation, University of Science and Technology of China, Hefei 230027, China; zm8218@mail.ustc.edu.cn (M.Z.); daiyc@mail.ustc.edu.cn (Y.D.); jrchu@ustc.edu.cn (J.C.); 2 School of Mechanical and Automotive Engineering, Anhui Polytechnic University, Wuhu 241000, China; wanghai@ahpu.edu.cn

**Keywords:** atmospheric pressure plasma microjet, polyurethane, maskless surface modification, gelatin, covalent grafting

## Abstract

A localized maskless modification method of polyurethane (PU) films through an atmospheric pressure He/O_2_ plasma microjet (APPμJ) was proposed. The APPμJ system combines an atmospheric pressure plasma jet (APPJ) with a microfabricated silicon micronozzle with dimension of 30 μm, which has advantages of simple structure and low cost. The possibility of APPμJ in functionalizing PU films with hydroxyl (–OH) groups and covalent grafting of gelatin for improving its biocompatibility was demonstrated. The morphologies and chemical compositions of the modified surface were analyzed by scanning electronic microscopy (SEM), Raman spectroscopy, and X-ray photoelectron spectroscopy (XPS). The fluorescent images show the modified surface can be divided into four areas with different fluorescence intensity from the center to the outside domain. The distribution of the rings could be controlled by plasma process parameters, such as the treatment time and the flow rate of O_2_. When the treatment time is 4 to 5 min with the oxygen percentage of 0.6%, the PU film can be effectively local functionalized with the diameter of 170 μm. In addition, the modification mechanism of PU films by the APPμJ is investigated. The localized polymer modified by APPμJ has potential applications in the field of tissue engineering.

## 1. Introduction

With the development of tissue engineering, the interaction between biomaterials and cells has drawn much attention [1,2,3]. Advanced biomaterials should promote cell responses such as adhesion and spreading [4]. Polyurethane (PU) is a preferred biomaterial for many different applications, such as artificial heart or artificial blood vessels, because of its excellent physical and mechanical properties [5,6]. Nevertheless, PU with nonideal biocompatibility could result in low cell affinity and affect the cell adhesion onto its surface, which could limit its extensive applications. Therefore, it is important to modify PU with biocompatible components, such as gelatin [7], chitosan [8], and other extracellular matrix proteins [9] for improving its biocompatibility. 

The surface modification methods of the biomaterial can be attributed to the followings: wet chemical [10], ultraviolet light irradiation [11], ozone treatment [12], nonthermal plasma treatment [13], etc. However, wet chemical methods need to use toxic reagents which can easily lead to environmental pollution [14]. Besides, a major drawback of ultraviolet light irradiation and ozone treatment is that they tend to enhance polymer degradation [15,16]. Nonthermal plasma treatment has a wide range of applications due to the advantages of being solvent free, treating complex shaped surfaces, and not affecting the mechanical properties of the polymer [17]. However, the conventional low pressure plasma surface modifications need complex and expensive vacuum systems [18]. Atmospheric pressure plasma is generated in ambient air, and has been considered as one of new key technologies in the field of surface treatment. Several plasma sources have been designed for surface processing of materials, such as dielectric barrier discharges (DBDs) [19,20] and atmospheric pressure plasma jets (APPJs) [21,22]. The typical electrode shapes of DBD sources are mainly parallel plate and cylinder. The treated material needs to be placed between the electrodes, so the space is limited. APPJs are generated in open space rather than in confined gaps, therefore, there is no limitation on the size and structure of the object to be treated. In recent years, more and more attention has been focused on the local modification of biomaterials to improve cell adhesion and migration, which has potential applications in cell patterning [23,24], biosensors [25], etc. Nevertheless, it inevitably requires masks to obtain micropatterns when using the APPJ to modify the biomaterial surface, which not only increases costs, but also limits the flexibility of fabrication.

A large amount of effort has been made by researchers in recent years to solve those problems. An atmospheric pressure plasma microjet (APPμJ) generated by various types of discharge mechanisms has been developed. The characteristic size of APPμJ can reach micrometers or even nanometers at atmospheric pressure [26,27]. It provides a method for micro/nano-scale surface modification without a mask. In our previous work, a novel APPμJ-based material processing system has been proposed and successfully applied for maskless localized etching of photoresist [28]. Different from other APPμJ structures, our APPμJ has a microfabricated micronozzle which is attached to the outlet of the millimeter scale quartz tube. The separated structure is convenient for assembly and replacement, and it is therefore easy to obtain plasma microjets with different diameters and their arrays, which could improve the flexibility and solve the problem of low processing efficiency of a single jet. To our knowledge, only a few researchers have studied the local surface modification of biomaterial by APPμJ for covalently coupling proteins [29,30], which may have great potential in the field of biomedicine. The aim of our work is to achieve maskless micropatterning for surface grafting gelatin and to optimize the plasma process parameters. The realization of this method may provide potential applications for future cell adhesion and migration behavior research on the modified surface. In this work, a novel maskless microplasma modification approach to achieve the localized modification of the PU films for gelatin immobilization was proposed. When PU film is exposed to the He/O_2_ APPμJ, plasma injected from a 30 μm micronozzle introduces hydroxyl (–OH) groups onto film surface, followed by modification with organosilanes. Then, the glutaraldehyde can react with organosilanes to form stable self-assembled monolayers. The glutaraldehyde is subsequently used to immobilize gelatin by covalent bond, which is extensively used to improve the biocompatibility of the PU film. In addition, the influence of gas composition and treatment time were investigated. The results show that the effective functionalized area is approximately 170 μm in diameter when treated with 0.6% gas mixture plasma for 5 min. Electrical characteristics and optical emission spectral (OES) of APPμJ were also investigated. X-ray photoelectron spectroscopy (XPS), Raman spectroscopy, fluorescence microscopy, and scanning electronic microscopy (SEM) were used to characterize the surface chemical composition and morphology changes.

## 2. Materials and Methods

### 2.1. Material

Polyurethane (PU, Bayer AG, Leverkusen, Germany) was used without further purification. Glutaraldehyde (25%, Grade I) and (3-aminopropyl) triethoxy silane (APTES, 99%) were purchased from Sigma-Aldrich Pte. Ltd. and used as received. FITC Conjugated Gelatin was purchased from Thermo. Ltd. (Waltham, MA, USA). Phosphate buffer saline (PBS) was purchased from HyClone.

### 2.2. Fabrication of Smooth PU Films

A portion of 0.5 g polyurethane particles were dissolved in a solution of 5.0 mL tetrahydrofuran (THF) and stirred rapidly by magnetic stirring until it was completely dissolved. A planar polymer film was received by dropping the solution on the glass. The remaining tetrahydrofuran was evaporated until an approximately 150 μm thick film was formed. Finally, the prepared film was placed into a vacuum oven and dried at room temperature to constant weight.

### 2.3. Atmospheric Pressure He/O_2_ Plasma Microjet

**Figure 1:**
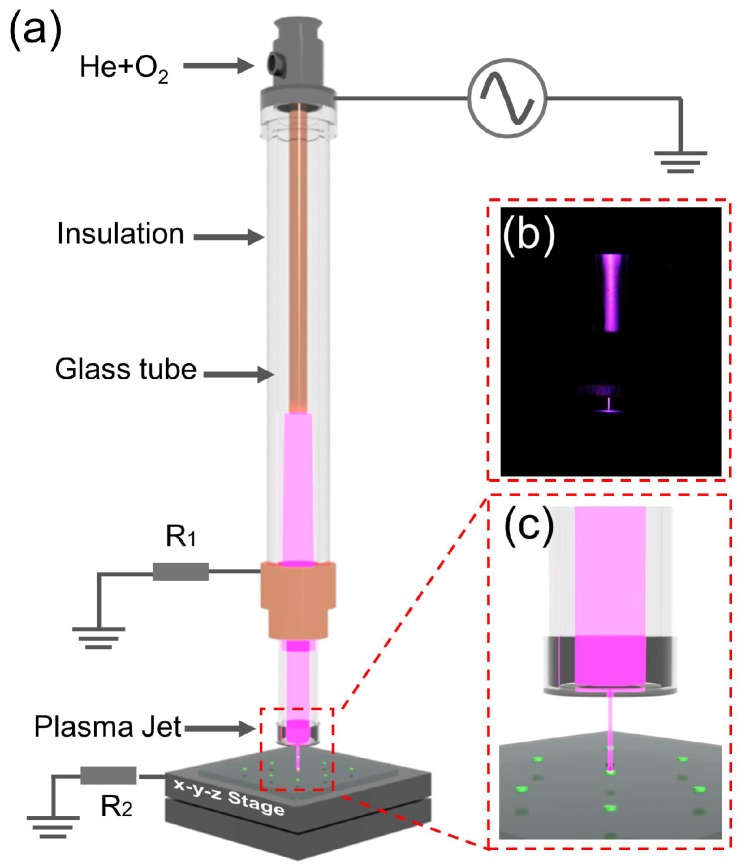
(**a**) The schematic of the atmospheric pressure plasma microjet (APPμJ) system; (**b**) photograph of the plasma microjet ejected from a 30 μm silicon micronozzle; (**c**) local magnified image of the APPμJ.

The schematic diagram of the APPμJ system is shown in Figure 1a. Figure 1c shows a local magnified image of the APPμJ. This system includes an APPJ based on the dielectric barrier discharge (DBD) principle, an inverted pyramid silicon micronozzle, XYZ axis linear stage. The APPJ is composed of a quartz tube, a Teflon tube, a high-voltage (HV) electrode, and a grounded electrode. The discharge of this pin-ring electrode structure is intense, resulting in lots of active plasma particles [31]. The length of the quartz tube is 120 mm, and the inner and outer diameters are 3 and 5 mm, respectively. A copper rod with a diameter of 2 mm and length of 80 mm is mounted inside the quartz tube, which works as a high voltage electrode. A hollow Teflon cylinder is wrapped around the glass tube as an insulating layer. A ring-shaped copper ground electrode is fitted around the Teflon tube, which is placed at a distance of 30 mm from the tip of the high-voltage electrode and 40 mm away from the edge of the quartz tube. An inverted pyramid silicon micronozzle with base dimension of 30 μm is attached to the outlet of the quartz tube. A detailed description of the fabrication process of the micronozzle is given in the next paragraph. The distance between micronozzle and PU surface is fixed at 1.0 mm. When the plasma is generated in the tube, it can be delivered from the micronozzle to the surface of the PU film, focused on the center. Figure 1b shows a photograph of the plasma microjet ejected from a silicon micronozzle taken by digital camera.

**Figure 2:**
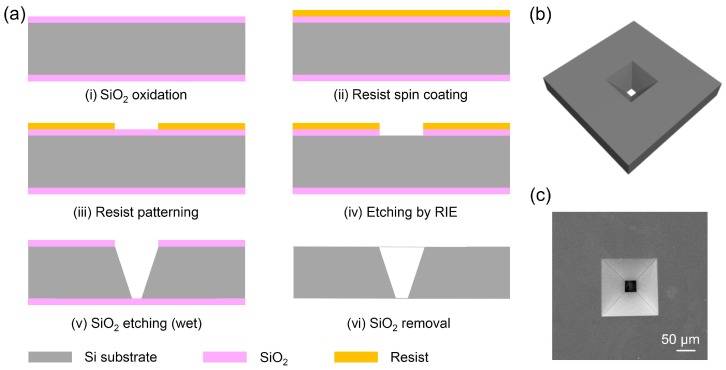
(**a**) Schematic diagram of the inverted pyramid silicon micronozzle fabrication procedure; (**b**) The diagram of the silicon micronozzle; (**c**) Scanning electronic microscopy (SEM) images of the silicon micronozzle.

Figure 2a shows the fabrication procedure of the inverted pyramid silicon micronozzle. It was fabricated on a 150 ± 10 μm thick p-Si (100) wafer by microfabrication technologies. The process begins with a silicon wafer produced by conventional oxidation (i), photolithography (ii, iii), and reactive ion etching (RIE, iv). The silicon dioxide was then used as the mask, and the inverted pyramid micronozzle with the upper and lower dimensions of 242 μm and 30 μm was obtained by conventional anisotropic wet etching with KOH (v). Finally, the protective layer of silicon dioxide was removed with hydrofluoric acid (vi). The schematic diagram and SEM images of the micronozzles are shown in Figure 2b,c, respectively.

### 2.4. Modification of Functionalized PU Films with Gelatin

**Figure 3:**
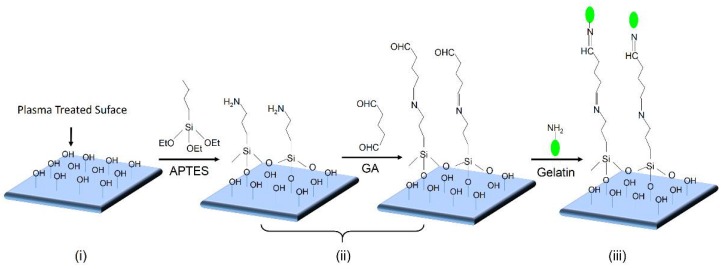
Schematic diagram of the three-step surface modification protocol.

The surface modification protocol is illustrated in Figure 3. Three steps are involved: (i) He/O_2_ plasma microjet treatment; (ii) (3-aminopropyl)triethoxy silane APTES and glutaraldehyde (GA) graft polymerization; and (iii) gelatin immobilization.

The prepared smooth PU films were treated by a helium and oxygen gas mixture APPμJ for hydroxyl group (–OH) functionalization [32]. The functionalized hydroxyl groups on PU films make covalent bonding to biomolecules possible, such as to protein and gelatin [33,34]. The PU film was placed on the platform and subjected to the microplasma for different process parameters to obtain the optimum result. Surface-activated PU films were then immersed into 150 mL 0.5% APTES aqueous solution for 30 min to form APTES-PU [35,36], after drying in oven at 50 °C, it was then immersed into 150 mL 2.0% GA solution for 30 min to form GA-APTES-PU, which was used for gelatin immobilization. For studying the possibility of connecting biomolecules on the polymeric surface with the organosilane as intermediates, we used a fluorescently labeled gelatin with a molecular weight of 10,000 Da. In this way we can detect the eventual gelatin immobilization by fluorescence microscopy. Thirty ul of 1 mg/mL fluorescein isothiocyanate (FITC)-Gelatin solution were manually deposited onto the as-prepared film subjected to aldehyde groups of GA overnight to form gelatin-immobilized PU films, referred to as gelatin-GA-APTES-PU. Subsequently, unreacted gelatin was removed by washing extensively with PBST solution (0.5% Tween-20 in PBS buffer), PBS solution, and then distilled water for 3 times, respectively.

### 2.5. Diagnostic Methods

The APPμJ is driven by the AC sinusoidal resonant high voltage power source (CTP-2000 K, Nanjing Suman Electronics Co., Nanjing, China) of ±7.0 kV amplitude at a frequency of 10 kHz. The discharge is produced in a glass tube with 1000 sccm helium and 6 sccm oxygen mixed gases. The flow rates of helium and oxygen are controlled by mass flow meters, respectively. The voltage of discharge is measured by a high voltage probe (Tektronix, Inc., P6015A, 1000:1, Beaverton, OR, USA), and the discharge current of plasma in the glass tube (I1) and plasma microjet (I2) were obtained by measuring the voltage by 10 Ω non-inductive resistors, R1 and R2, respectively. The electrical behavior was monitored via a Tektronix digital oscilloscope DPO-3014, and the optical emission of the discharge was collected by an optic spectrometer through an optical fiber (Avantes, AvaSpec-2048-4, wavelength: 250–950 nm, resolution: 0.12 nm, Apeldoorn, The Netherlands). The fiber integrated with the collimating lens was placed at the side of the microjet and collected optical emission from plasma. The morphologies of modified PU films were observed by optical microscopy (BA310Met-T, Motic) and SEM (Zeiss Evo 18). XPS analysis was performed by X-ray photo-electron spectrometry (Thermo ESCALAB 250, Thermo Fisher Scientific, Waltham, MA, USA) with an excitation source of Al Ka radiation (*hυ* = 1486.6 eV). The X-ray spot size was 500 μm. Raman measurements were performed using a 785 nm laser and an Andor Shamrock SR-500iA spectrometer (Andor Tech, Belfast, Northern Ireland) equipped with a charge-coupled device (CCD) camera (DV420A-OE, Andor Tech) connected to PC. A 60× water immersion objective lens was employed with the confocal microscope to focus the laser, which was focused (50 mW) on the sample over an exposure time of 10 s.

## 3. Results and Discussion

### 3.1. Electrical Characteristics of APPμJ 

**Figure 4:**
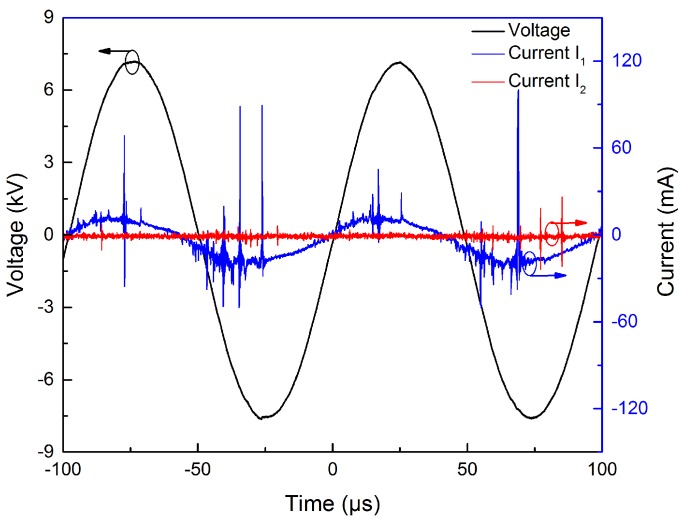
Typical current–voltage waveforms of the atmospheric pressure He/O_2_ plasma microjet (APPμJ).

Figure 4 shows the typical waveforms of applied voltage and current characteristic of APPμJ. According to the waveforms, multi-spikes are observed in the current waveforms (I1) during both positive and negative half cycles of applied voltage. The life of each current pulse is several tens of nanoseconds. We speculate these current peaks correspond to the typical filament discharge between power and ground electrodes through the quartz discharge tube. The discharge current of the plasma microjet (I2) has several multi-spikes. During these current peaks, plasma bullets ejected from silicon nozzle are irradiated onto the PU film surface [37,38]. 

### 3.2. The Optical Emission Spectral Characteristics of APPμJ

**Figure 5:**
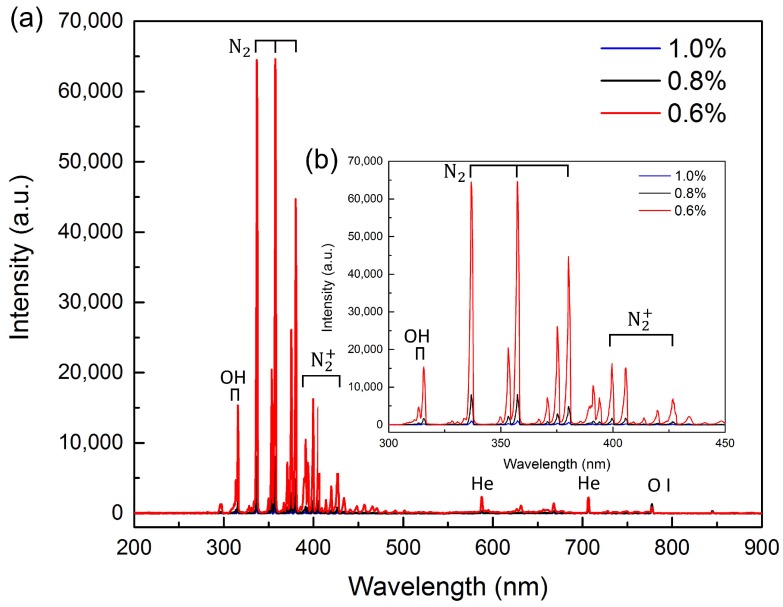
Optical emission spectra of APPμJ under 0.6% O_2_–He gas mixture. (**a**) Optical emission spectra of the APPμJ under the same applied voltage of ±7.0 kV; (**b**) Different volumes of oxygen are added to obtain the optimum parameters.

Figure 5a shows the optical emission spectra of the APPμJ under the same applied voltage of ±7.0 kV. The spectrum shows that there are strong nitrogen molecular lines as well as a few helium and oxygen atomic lines. Nitrogen species arise because the plasma is ejected into the air where its energetic electrons and He metastables ionize and excite air molecules [39]. The emission spectrum clearly indicates that OH (306–309 nm), O (777 nm), and He (587, 707 nm) exist in the plasma microjet. The lines 337, 357, and 380 nm represent the second positive system of nitrogen molecules. The lines of the first negative system of N_2_^+^ at 391 and 427 nm are also visible in the spectrum [40]. In order to study the effect of the oxygen concentration on oxygen-containing functional groups, different volumes of oxygen are added to obtain the optimum parameters, as shown in Figure 5b. It is noted that with the oxygen ratio increasing from 0.6% to 1.0%, the intensity of reactive O atom and OH radicals decrease. The most important reason is that oxygen, as an electronegative gas, has high-electron affinity. When the seed electron density is reduced with the increase of oxygen ratio, weakening of the discharge can be observed [41]. The decreasing of the reactive species could affect the number of surface functional groups, which is in accordance with the fluorescence microscopy images. Details will be discussed in the following sections.

### 3.3. XPS Analysis

**Figure 6:**
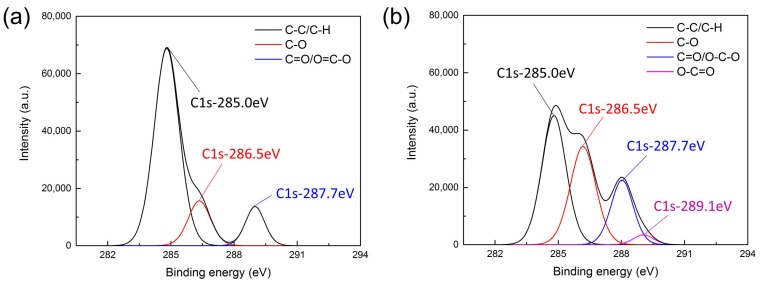
High-resolution C1s peak of (**a**) an untreated PU film and (**b**) a He/O_2_ plasma-treated PU film.

As show in Figure 6a,b, XPS measurements were performed on pristine and plasma treated samples to get an insight into the chemical composition of the PU surface. Based on the deconvoluted C1s peaks, the concentration of the different chemical bonds can be calculated and the obtained results are given in Table 1. It can be seen that the concentration of C–C and/or C–H bonds decreases after He/O_2_ plasma treatment, while the concentration of oxygen-containing groups (C–O, C=O, and O–C=O) strongly increases. During the treatment, we conjecture that metastables, reactive species, and UV and VUV photons in the plasma microjet will weaken and break the C–C and/or C–H bonds of the outermost PU film for surface activation [39,42]. As a result, the ratio of C–C and/or C–H bonds decreases. Oxygen, as a reactive gas, can generate the oxygen-containing functional groups and these groups can be bonded to the macromolecule chain directly, changing the chemical composition of the film surface. Moreover, the plasma itself consists of charged species such as electrons and He metastables which can ionize the oxygen and H_2_O in surrounding air to create more exited species, such as O^+^, OH^+^, O^−^, O^∗^, and N_2_^+^ [43,44]. Therefore, the increase of O indicates that oxygen-containing polar groups are formed on the surface, which is important for biomolecule immobilization. Besides, an increase of N is mainly due to the ionization of the nitrogen in the ambient air, however, it does not react with organosilanes to form stable self-assembled monolayers and does not affect the grafting of gelatin.

Meanwhile, the C1s peak of the XPS spectra was also investigated in detail to evaluate which oxygen-containing functional groups have been incorporated on the PU surface by plasma treatment. As shown in Figure 6a, the C1s peak of the untreated PU film can be decomposed into three distinct peaks, i.e., C–C/C–H (285.0 eV), C–O (286.5 eV), and C=O/O–C–O (287.7 eV) [45]. Figure 6b shows the C1s peak of the PU film after He/O_2_ plasma treatment, the peak at 285.0 eV sharply decreases while the peaks at 286.5 and 287.7 eV increase. The increase of the peak at 286.5 eV is due to the generation of hydroxyl groups which are important for gelatin immobilization. Moreover, a new peak at 289.1 eV, which can be attributed to O=C–O groups, appears after plasma treatment.

According to the XPS results, the surface of PU films have been locally functionalized with oxygen-containing functional groups.

### 3.4. Surface Morphology of PU Film Functionalized by APPμJ and Modified by Gelatin

**Figure 7:**
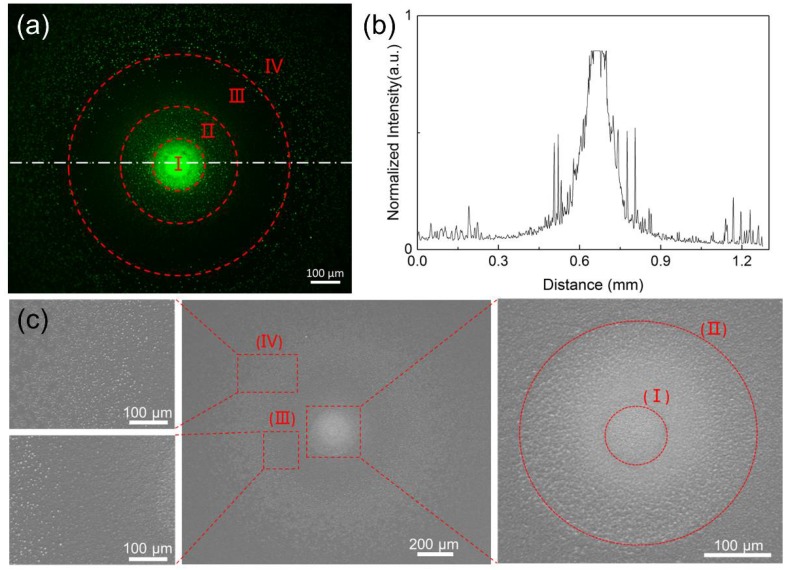
(**a**) Fluorescence microscope image of the gelatin immobilized PU film which can be divided into four regions (I–IV) depending on the fluorescence intensity; (**b**) Normalized fluorescent intensity profiles of the dash-dotted line in the fluorescence microscope image; (**c**) SEM images of plasma treated PU film.

Figure 7a shows the micropatterns of FITC-gelatin on PU film modified by APPμJ for 5 min. The applied voltage is ±7.0 kV, the helium gas flow rate is 1000 sccm, and oxygen gas is 6 sccm. Figure 7b is the normalized fluorescent intensity profiles of the dash-dotted line in the fluorescence microscope image. We find an interesting phenomenon that the fluorescent intensity of the functionalized area is not uniform. It is obvious that four different regions can be observed from the center to the outside area, which are defined as I to IV. The center region (170 μm) shows the strongest fluorescent intensity, followed by region II (390 μm) and region IV, indicating that the center region has the best immobilization efficiency of gelatin. In the process of modification, large amounts of high energy He atoms or reactive species in the plasma microjet accelerate the surface physical bombardment of the film, which induces a variation in the surface roughness. As shown in Figure 7c, the roughness in the central area is higher than in region II because of the different distribution of the active particles in the microjet [46,47]. Along the expansion of rings, the diameter of region III is about 750 μm. The surface morphology of this region is relatively smooth and the fluorescence intensity is the weakest. It means that there are no effective functional groups in region III. The result is in accordance with the Raman spectroscopy analysis in Section 3.5. This phenomenon is due to the diffusion effect when the plasma is transferred to the surface of the sample and the interactions between the plasma microjet and the surrounding air [39]. Region III should be the transition area between the core area and the peripheral area of the plasma propagating along the film surface. We conjecture that the smooth surface is attributed to the influence of airflow [48]. The mechanism of surface modification still deserves further study. The diameter of the outermost region IV is about 1200 μm and the roughness and the fluorescent intensity of this area are relatively lower than the central area because the chemical species in the expansion plasma differed from the center towards the outside [47,48,49,50].

### 3.5. Raman Spectroscopy Analysis

**Figure 8:**
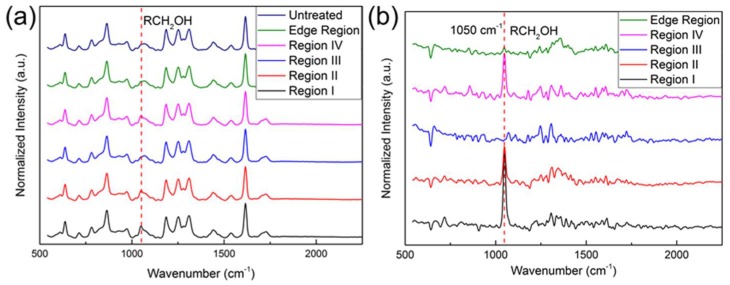
Raman spectra of the plasma-treated PU film. (**a**) The Raman spectra of 5 different regions (regions I–IV and the edge region) of the plasma-treated PU film and untreated PU film; (**b**) Further processed Raman signals of the plasma-treated PU film.

To further verify our inference, we performed Raman spectroscopy analysis. As shown in Figure 8a, the Raman spectra of 5 different regions (regions I–IV and the edge region) of the plasma-treated PU film and untreated PU film were measured. From the Figure 8a, we can see the primary alcohol (RCH_2_OH) peak at 1050 cm^−1^ [51]. In order to better understand the difference between the peaks of different regions, we further processed these Raman signals. The Raman signals of region I–IV and the edge region on the plasma-treated sample were subtracted from the Raman signal of the untreated region, resulting in Figure 8b. There is a distinct peak at 1050 cm^−1^. The peak of region I is the strongest, followed by region II and region IV. Region III is almost similar to the edge region, and no obvious peak is found. The different fluorescence intensities of the treated area were attributed to the different hydroxyl distributions on PU film surface. This result is consistent with our fluorescence result and also verifies our hypothesis.

### 3.6. Influence of O_2_ Flow Rate and Plasma Treatment Time

**Figure 9:**
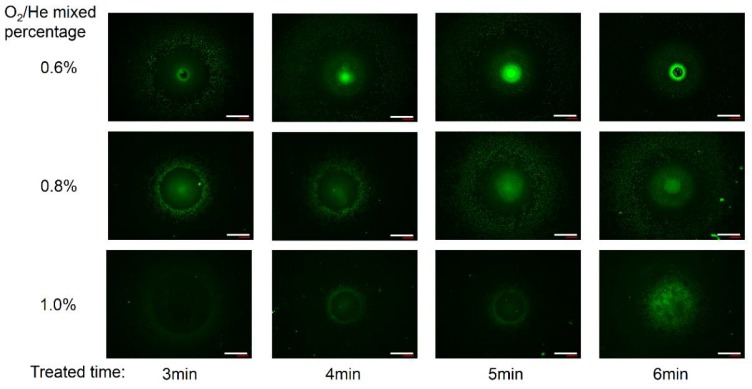
Fluorescence microscope images of the gelatin immobilized PU films which were treated by plasma microjet with different O_2_/He mixed percentages (0.6–1.0%) for different times (3–6 min). Scale bar: 200 μm.

For further evaluation of the functional area by the plasma-assisted process, we analyzed the effects of other processing parameters. Figure 9 shows that the fluorescence images of the functional area varies with processing time and oxygen percentage. Under the same treatment time, as the oxygen flow rate increases from 6 sccm to 10 sccm, the fluorescence intensity of the region I and II was gradually decreased. The increase of the oxygen gas flow rate could lead to a significant decrease in the density of oxygen radicals. It is attributed to the recombination of active species [52], which is in accordance with the optical emission spectrum analysis. Beyond that, the fluorescence intensity of region IV was also decreasing. The additional oxygen might absorb the electrons and quench the metastable He atoms [41]. The decrease of energetic electrons and metastable He atoms makes the physical bombardment effect weakened, thus decreasing the roughness.

Under the same O_2_ flow rate, the fluorescence intensity increases gradually as the processing time increasing. Analyzing from the intensity distribution of fluorescence images, when the processing time is 4 to 5 min with the oxygen percentage of 0.6%, the PU film can be effectively locally functionalized with hydroxyl groups. When the processing time increases to 6 min, the central region I of the film surface was seriously damaged, which caused no gelatin grafting on this region. Besides, when the oxygen percentage is 0.8% or 1.0%, the concentration of fluorescence intensity of regions I and II was slightly increased as the processing time increasing, which indicated the functionalized efficiency is improved slightly. 

## 4. Conclusions

In this paper, a method for localized maskless modification of PU films by using an APPμJ was proposed. We demonstrated the possibility that He/O_2_ plasma is able to functionalize PU films for gelatin immobilization. Due to the different spatial distribution of oxygen-containing functional groups and the interaction between microplasma and ambient air, different areas of the PU film were grafted with gelatin with different fluorescence intensity. Finally, the effects of plasma processing time and oxygen flow rate on the surface functional results of the PU films were discussed. When the processing time is 4 to 5 min with the oxygen percentage of 0.6%, the PU film can be effectively locally functionalized with the diameter of 170 μm. We found that the ratio of O_2_ flow had a great influence on the functional results, and that higher O_2_ flow tended to weaken the discharge intensity and reduce the degree of functionalization. We believe that the localized maskless modified polymer surfaces by APPμJ could offer opportunities for applications in the tissue engineering, biosensing, and biomedical fields. In the future work, we will combine the silicon micronozzle arrays with scanning technology to realize localized maskless micropatterning of biomaterials.

## Figures and Tables

**Table 1:** Atomic composition and concentration of chemical groups on the untreated sample and plasma-treated PU sample. (He/O_2_ microjet, treatment time: 300 s, helium flow rate: 1000 sccm, oxygen flow rate: 6.0 sccm, applied voltage: ±7.0 kV, nozzle–polymer distance: 1.0 mm).

Sample	O/C Ratio (at%)	C–N (%)	C–C/C–H (%)	C–O (%)	C=O/O–C–O (%)	O–C=O (%)
Untreated	29.18	3.41	71.22	14.52	10.84	0
Treated	35.23	19.40	34.93	27.86	15.60	2.21
